# Perception of interpersonal distance and social distancing before and during COVID-19 pandemic

**DOI:** 10.1038/s41598-024-55218-y

**Published:** 2024-02-25

**Authors:** Nur Givon-Benjio, Hili Sokolover, Idan M. Aderka, Bat-Sheva Hadad, Hadas Okon-Singer

**Affiliations:** 1 School of Psychological Sciences, University of Haifa, Haifa, Israel; 2 Department of Special Education, University of Haifa, Haifa, Israel; 3 The Integrated Brain and Behavior Center (IBBRC), University of Haifa, Haifa, Israel

**Keywords:** COVID-19, Interpersonal distance, Distance preference, Distance perception, Psychology, Human behaviour, Health policy, Public health

## Abstract

Since COVID-19 is easily transmitted among people in close physical proximity, the focus of epidemiological policy during the COVID-19 crisis included major restrictions on interpersonal distance. However, the way in which distance restrictions affected spatial perception is unclear. In the current study, we examined interpersonal distance preferences and perceptions at three time points: pre-pandemic, early post-pandemic, and late post-pandemic. The results indicate that following the pandemic outbreak, people perceived others as farther away than they actually were, suggesting that the distance restrictions were associated with an enlargement of perceived interpersonal distance. Interestingly, however, people maintained the same distance from one another as before the outbreak, indicating no change in actual distance behavior due to the risk of infection. These findings suggest that COVID-19 was associated with a change in the way distance is perceived, while in practice, people maintain the same distance as before. In contrast, COVID-related anxiety predicted both a preference for maintaining a greater distance and a bias toward underestimating perceived distance from others. Thus, individuals who were highly fearful of COVID-19 perceived other people to be closer than they actually were and preferred to maintain a larger distance from them. The results suggest that subjective risk can lead to an increased perception of danger and a subsequent change in behavior. Taken together, even when behaviors should logically change, the decision-making process can be based on distorted perceptions. This insight may be used to predict public compliance.

## Introduction

### Social distancing during COVID-19

The COVID-19 pandemic was first detected in China at the end of 2019 and spread rapidly across the globe. People were infected mainly when they were in physical proximity of others. Therefore, epidemiological policy focused on increasing the interpersonal distance people maintain from one another. Measures to enforce this policy included restrictions on activities, distributing information through media to increase public awareness and compliance, and imposing fines for violation of the restrictions. Extreme measures to ensure distancing were also utilized—namely personal quarantine and general lockdown. The policy of enforcing distance during the COVID-19 pandemic has no match in its extent, duration, and costs^1^. The mandated increase in interpersonal distance was directly linked to a decrease in infection and mortality rates^2–4^, thus highlighting the importance of examining mechanisms that may predict and facilitate increased interpersonal distance.

At the onset of the pandemic, it was initially assumed that maintaining a 2-m distance would be sufficient to prevent infection^5^. However, it soon became clear that infection could occur even at 8 m, depending on factors such as the type of interaction (e.g., talking) and room ventilation^6,7^. Consequently, the implementation of additional safety measures became crucial, leading to public instructions to adopt all safety measures to reduce the chances of virus transmission^8^. However, a growing concern emerged that emphasizing one safety measure, such as wearing masks, might create a false sense of security, resulting in a reduction in the adherence to other safety measures, such as social distancing^9–11^. This phenomenon is referred to as the risk compensation effect, a concept existing in the literature before the pandemic, defined as the use of one safety measure leading to a reduction in the usage of other safety measures. For example, prior studies have suggested that mandatory safety-belt use increases risk-taking behaviors among drivers due to a false sense of safety^12–14^. However, recent findings suggest only weak to no evidence for such an effect during COVID-19^15–18^. Instead, there is stronger evidence suggesting that implementing one COVID-related safety measure, such as masks, increases the usage of other safety measures, such as social distancing and personal hygiene^19,20^. Therefore, the significance of maintaining interpersonal distance persisted throughout the pandemic, driven by both actual risk factors and psychological considerations.

Following the onset of the pandemic, studies examining interpersonal distance during COVID-19 indicated that requiring people to maintain a large physical distance during the pandemic may have changed what is considered ‘appropriate’ interpersonal distance^21^. Furthermore, COVID-related loneliness was associated with a preference for proximity^22^. Welsch, Wessels, Bernhard, Thönes, and von Castell (2021)^23^ collected data at three time points during the acute stages of the COVID-19 pandemic. Their findings suggest that at the beginning of the pandemic, people rapidly increased their preferred distance and that this effect lingered after the pandemic^23^. Yet, other evidence suggests that the preference for interpersonal distance is associated with an individual’s subjective fear of being infected with coronavirus and not with the actual objective risk of infection^24^. The role of risk in regulating behavior during COVID-19 is also evident in findings indicating that even in a virtual environment, people chose to maintain a greater distance from people who were not wearing face masks^25–27^. Despite extensive research examination, COVID-related changes in interpersonal distance are still largely inexplicable. Given the crucial role interpersonal distance plays in managing the pandemic, shedding light on the processes underlying distance preference is of major importance.

### Biases in perception of physical distance

In a related vein, a growing body of evidence suggests that perception can be altered by top-down processes, such as emotions^28^, actions^29^, and motivations^30^, with studies pointing out a specific influence on the perception of physical distance. For instance, the act of wearing a heavy backpack can make us perceive distance as greater^31^, as can throwing a heavy ball^32^. Furthermore, there is also accumulating evidence for influences of motivation (desires, needs, values, etc.) on perception. For example, desirable objects, such as a bottle of water when one is thirsty, are perceived as closer compared to less desirable objects^33^. Similarly, research has indicated that a desirable location can appear closer in perceived distance compared to an equidistant undesirable location^34^. Research into the influence of emotions on perception has shown that when individuals view a threatening person, they tend to perceive that person as physically closer compared to viewing someone with a neutral affect or someone who evokes another strong negative affective response, such as disgust^35^. These findings align with prior research indicating that individuals with social anxiety disorder, characterized by heightened anxiety around strangers, tend to perceive strangers as closer in physical proximity compared to friends, neutral stimuli, and actual physical distances^36,37^. Studies examining the peripersonal space—the area surrounding our body that is within our reach^38^ have also revealed that threatening stimuli located near the body can lead to a reduction in the size of this reachable space^39,40^. Furthermore, it has been proposed that the direction of perceptual bias—whether the distance is underestimated or overestimated—depends on the intricate interplay between an individual's motivation to approach or avoid, the level of effort required for a particular action, and the emotional valence of the stimulus (i.e., whether it is perceived as rewarding or threatening^41,42^). During the COVID-19 pandemic, one study found that restrictions on physical distance led to a reduced sense of peripersonal space^43^. In the context of interpersonal distance perception, a recent study indicated that interpersonal distance from a third-party perspective (i.e., distance between two strangers) was underestimated for close proximity (50 cm and 90 cm) and overestimated for large distances (150 cm)^44^. Nonetheless, to the best of our knowledge, this was the only study that examined changes in perceived interpersonal distance in the context of COVID-19. Moreover, interpersonal distance has never been examined from a first-person perspective (i.e., estimations of how far other people are from us). Furthermore, inconsistencies regarding changes in distance preference in the wake of COVID-19 highlight the need for further examination.

### The current study

The pandemic created a sort of “natural experiment” where people's behavior and perceptions were impacted by sudden and significant changes in their daily lives. The implementation of social distancing measures offered a unique opportunity to examine how external restrictions can influence human behavior. However, the effects of these restrictions on interpersonal distance preference and perception during COVID-19 are not yet clear. Therefore, the aim of this current study was to investigate COVID-related changes in distance preference and perception as a function of objective measures of COVID-19 risk (infection rate) and of subjective measures of COVID-related anxiety. Furthermore, since COVID-19 pandemic itself was an evolving event, we also looked at the changes in distance preference and perception as a function of the passage of time since the outbreak. We hypothesized that objective risk of COVID-19 would be associated with an increase in the distance people prefer to maintain from one another. Since distance perception has never been examined in the context of COVID-19, we proposed no hypotheses concerning changes in distance perception and examined this as exploratory research. Based on the literature, we hypothesized that subjective COVID-related anxiety would be associated with a preference for maintaining a greater distance from others. Furthermore, based on previous findings regarding the influence of fear on distance estimation e.g.,^36,37^, our hypothesis was that highly fearful individuals would exhibit a tendency to underestimate the distance from others.

## Method

### Data availability statement

Materials and datasets generated during the current study are openly available at the project’s Open Science Framework page repository (https://osf.io/tcg3v/; FOR THE BLIND REVIEW: https://osf.io/tcg3v/?view_only=a4976fa9cb724bcf8b835ed8ec8edfdc ).

### Participants

Data were collected at three time points: (1) *Pre-pandemic (2018–2020):* The sample included 87 participants: 44 men, 43 women (*M*_*age* =_ 26, *SD*_*age*_ = 7). Participants were sampled between December 2018 and early February 2020 (just a week before the outbreak of the pandemic in Israel). Sampling frequencies: 6.8% were sampled during 2018, 90.5% during 2019, and 2.7% during 2020. This sample was collected as part of a different project, and the data were analyzed post-hoc for the purposes of the current study. University of Haifa’s Ethics Committee’s approval number:/16,459. (2) *Earlier post-pandemic (2020–2022)*: The sample included 89 participants: 26 men, 63 women, 1 non/other; (*M*_*age* =_ 26, *SD*_*age*_ = 6.67). Participants were sampled between December 2020 (303 days after the outbreak of the pandemic in Israel) and June 2022 (844 days after the outbreak of the pandemic in Israel). Sampling frequencies: 2.2% were sampled during 2020, 37% during 2021, and 60.8% during 2022. This sample was collected as part of a different project, and the data were analyzed post-hoc for the purposes of the current study. University of Haifa’s Ethics Committee’s approval number: 358/20. (3) *Later post-pandemic (2022–2023)*: The sample included 84 participants: 27 men, 56 women, 1 non-binary/other; (*M*_*age* =_ 26, *SD*_*age*_ = 6.67); 7.1% reported being vaccinated at least four times, 58.3% reported being vaccinated three times, 26.2% reported being vaccinated twice, 2.4% reported being vaccinated once, and 6% reported not receiving any coronavirus vaccinations. Participants were sampled between November 2022 (1022 days after the outbreak of the pandemic in Israel) and March 2023 (1134 days after the outbreak of the pandemic in Israel). Sampling frequencies: 45% were sampled during 2022, and 55% during 2023. University of Haifa’s Ethics Committee’s approval number: 358/20 (with corrections approved). All studies were performed in accordance with relevant guidelines and regulations. Further, all participants have provided informed consent.

### Stimuli and design

#### The false interview task^36^ (Fig. 1)

**Figure 1:**
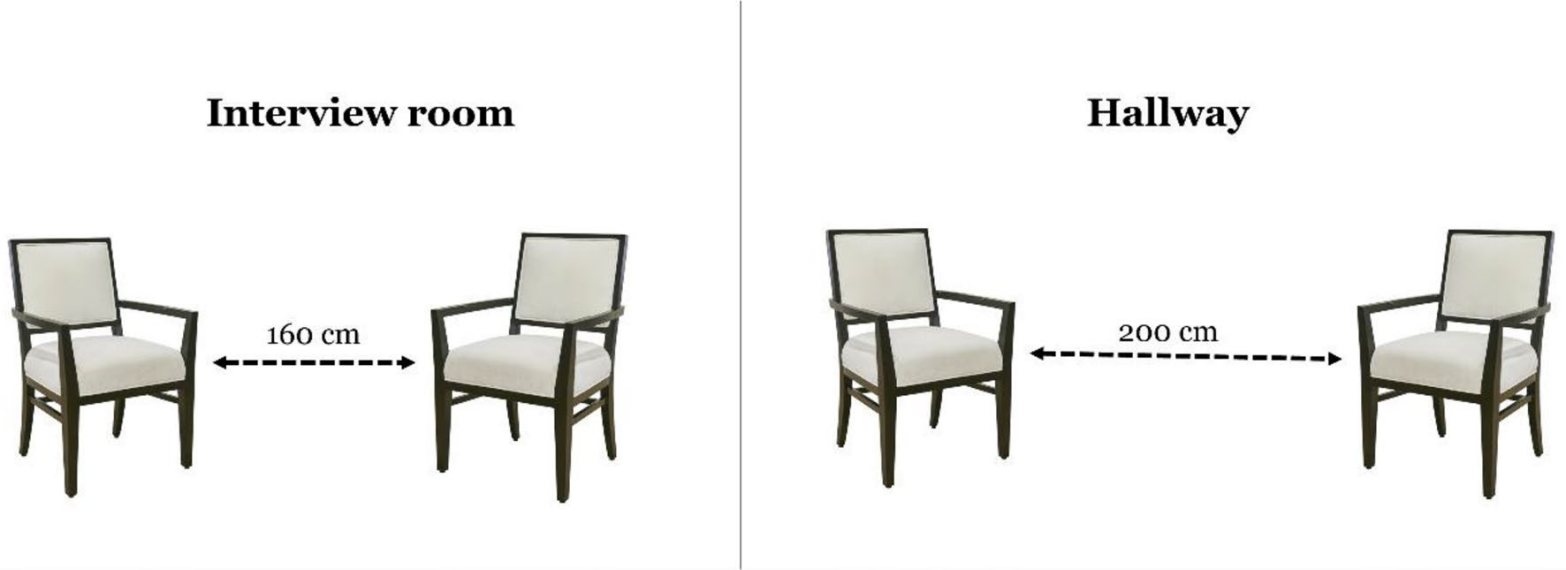
Example of the false interview task procedure. The left image depicts the distance between the chairs in the interview room (160 cm), which constitutes the preferred distance. The right image depicts the estimated distance in the hallway (200 cm). The distance perception bias is calculated as the difference between the two distances (+40 cm). In this example, the participant preferred to maintain a distance of 160 cm from the interviewer, but overestimated that distance by 40 cm, such that the interviewer was perceived as farther away.

In the false interview task, participants were told they are about to be interviewed by an unfamiliar interviewer. The experimenter asked the participant to follow him/her into the interview room, which was prearranged to contain only one chair (the interviewer’s chair), while the participant’s chair was missing. The experimenter then apologized and asked the participant to bring a chair from the adjacent room and to sit and wait for the interview to begin while the experimenter calls the interviewer. In reality, participants were only led to believe that an interview was about to take place, when in fact this task was only designed to measure the distance at which participants placed their chair from the interviewer’s chair as a measure of their preferred interpersonal distance.

After the participant had placed his or her chair (2–3 min), the experimenter returned to the interview room and informed the participant that the interviewer was running late. The participant was asked to move into the hallway, and the experimenter closed the door behind them. In the hallway, the experimenter set up two additional chairs, which were identical to the ones in the interview room, facing each other with zero distance between them. The participant was then instructed to arrange these two chairs so that the distance between them matched the distance between the chairs in the interview room. If participants had any questions about the purpose of this request, they were informed that they would receive a debriefing after the experiment, and no further explanation was provided. The distance at which the participant placed the additional chairs was measured, constituting the distance perception measure. The participant’s distance perception bias was calculated as the difference between the perceived distance and the preferred distance (subtracting distance preference from distance perception). A positive score represents overestimation of the distance, whereas a negative score represents underestimation of the distance. Therefore, the bias score was calculated as the participants’ ability to accurately replicate the distance between the chairs in the interview room (i.e., their preferred distance). After completing the task, participants were provided with a comprehensive debriefing. Furthermore, please note that a validation of this paradigm was conducted in our previous research^36,37^, where we demonstrated that participants had no suspicions regarding the deception and were unable to guess the true purpose of the experiment.

#### The preferred and estimated distance task (PED)

**Figure 2:**
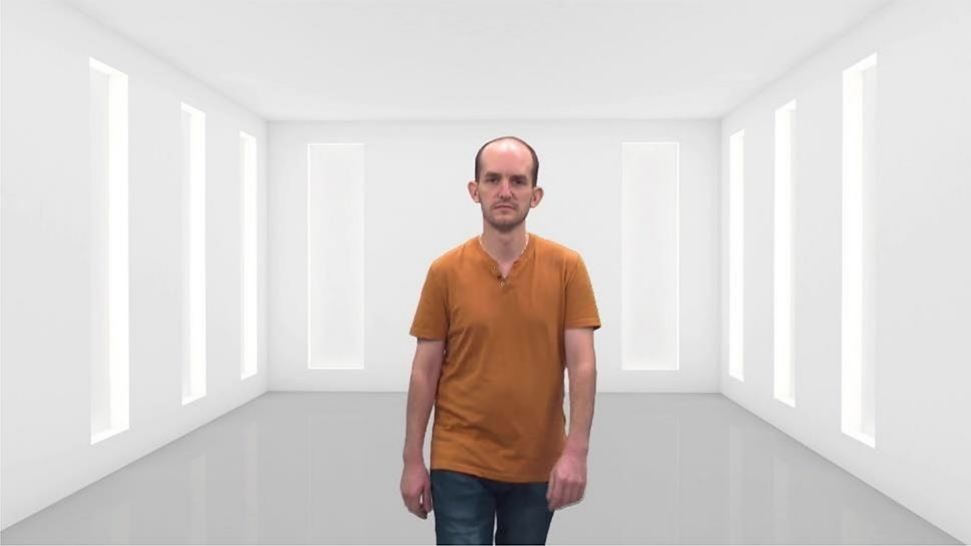
Example of the stimuli presented in the PED task. All stimuli and the complete task are available from the OSF open material link: https://osf.io/tcg3v/. All the models have provided signed permission to display their photo in scientific journals, as well as have consented to the use of their images in future researches.

The PED task includes two phases, presented sequentially. During Phase 1 (distance preference), participants are shown video clips depicting a stranger walking towards/away from them (i.e., the camera; see Fig. 2). All videos depict a model walking a total distance of 3.5 m. Participants were asked to stop the moving stranger at the preferred proximity by clicking the computer mouse in order to freeze the video. Participants were shown ten models (five male, five female). Each model was shown twice: once walking towards the participant and once walking away from the participant, resulting in 20 trials. The preferred distance was calculated in centimeters based on the following formula:

$$Distance \,moved\, in \,video - { }\left( {\frac{{Distance{ }\,moved{ }\,in{ }\,video }}{{Video{ }\,time}} \times The \,participant^{\prime}s \,answer} \right)$$

Please note that the 'distance moved in the video' was a constant value (350 cm), while the 'video time' varied between 8and 12 s due to differences in the model's speed across videos. For instance, in the case of a 10-s video, we determined the preferred distance by dividing the constant 350 cm (the total distance in the video) by the video duration (10 s), resulting in a rate of 35 cm per second. We then calculated the participant's preferred distance by multiplying this rate by their reaction time (e.g., 2 s), leading to a preferred distance of 70 cm. By subtracting this 70 cm from the total 350 cm, we arrived at the final preferred distance of 280 cm.

In Phase 2 (perception bias), each trial began with a picture depicting a stranger standing at some distance from the camera (for an example, see Fig. 2). After appearing for 1 s, the picture was replaced by a mask (i.e., blurred pixels of the scene) for 250 ms. Then, participants were shown a video clip depicting the same stranger. They were instructed to stop the video when the model reaches the exact same distance. Each model appeared at four different distances, and each distance appeared twice: once with the model walking backwards, and once with the model walking forwards. Participants completed 100 trials. The distance perception bias was calculated as the difference between the correct answer and the participant’s response, based on the following formula:

$$\left( {\frac{{Distance{ }moved{ }in{ }video }}{{Video{ }time}} \times Correct answer } \right) - { }\left( {\frac{{Distance{ }moved{ }in{ }video }}{{Video{ }time}} \times The participant^{\prime}s answer} \right)$$

Therefore, the bias score was calculated as the participants’ ability to replicate the actual distances in which the models stood. Using the same example, for a 10-s video, we divided 350 (total distance moved in the video) by 10 (total video time), giving us 35 cm. We then multiplied this by the participant's reaction time (e.g., 3 s), resulting in 105 cm. We also multiplied the correct answer (e.g., 2 s, representing the exact timestamp of the distance image) by 35 cm, yielding 70 cm. After transforming these values into distance units, we subtracted the correct answer (70) from the participant's answer (105), resulting in a score of 35. This score indicates that the participant estimated the distance from the stranger as 35 cm less than it actually was. Note that for reversed videos (depicting a stranger walking backward), the estimation bias score was multiplied by -1.

### Days since COVID-19 outbreak

This value represents the number of days that elapsed since the outbreak of the COVID-19 pandemic in Israel (where Day 0 represents the first confirmed case). In the earlier post-pandemic sample (two years following the outbreak), data were collected from Day 303 through Day 844. On five days during this period (5%) there was a lockdown (i.e., the third lockdown in Israel). In addition, on four days during this period (4.4%), the economy partially reopened following the lockdown, and on 82 days (90.1%) the economy fully reopened. In the later post-pandemic sample (the third year following the outbreak), data were collected from Day 1022 through Day 1134. The economy was fully open during that entire period.

### Infection rate (R value)

The R value is an approximation of the infection coefficient that represents the number of people each infected person is expected to infect. When the infection coefficient is greater than 1, the number of infected people may increase exponentially and growth of the pandemic may become uncontrolled. The R value is calculated every day as well as for a ten-day period. Data on the R value for every given day were obtained from Israel Ministry of Health public records. R value data were collected only for the second sample and not for the third because the Israel Ministry of Health stopped collecting this data during this period.

### Confirmed cases

This factor represents the change in daily confirmed cases of COVID-19. To mitigate daily fluctuations associated with external factors, such as variations in testing frequency (e.g., on rest days), the variable was calculated as follows: for each day, the daily difference in cases was determined by subtracting the total cases of the previous day from the current day. Subsequently, a 14-day rolling average was computed. The outcome serves to objectively measure risk, intended to replace the R value, which the Ministry of Health ceased to collect in the later post-pandemic phase. Please note that all available information, including the R value and confirmed cases (along with the rolling average calculation), is publicly accessible on the OSF repository link.

### Questionnaires

For the third sample, participants completed questionnaires examining subjective anxiety.

#### COVID-19 anxiety scale (CAS)^45^

The CAS is a seven-item questionnaire that measures levels of COVID-related anxiety. Items include statements on respondents’ level of fear and anxiety (e.g., “I feel anxious about COVID-19” and “I am afraid of being infected with COVID-19”). For each statement, participants were asked to indicate the degree to which each statement applied to them during the last few days, on a 4-point scale ranging from “not applicable to me” to “very applicable to me”. The internal consistency estimates also confirmed the good reliability of the questionnaire (α = 0.89, ω = 0.70)^45^.

#### The generalized anxiety disorder (GAD-7)^46^

The GAD is a seven-item questionnaire that measures respondents’ levels of generalized anxiety during the previous two weeks. Participants are given statements such as “Feeling nervous, anxious, or on edge” and “Worrying too much about different things” and are asked to report how often they were bothered by these problems, on a scale ranging from 0 (not at all) to 3 (nearly every day). A recent study that measured the internal consistency of the measurement during COVID-19 found that its psychometric properties were adequate (α = 0.99, ω = 0.90)^47^.

#### Demographic information

Participants were asked the following questions: number of vaccinations they had received (ranging from 0 to 4+), gender (woman/man/other), dominant hand (right/left/both), and age. Furthermore, after participants completed the questionnaires, we asked them whether they have any hypotheses regarding the goal of the study or what the study is intended to measure. This question was designed to control observer-expectancy effects.

### Procedure

The study included analyses of three separate samples, with slight procedural differences: (a) In the pre-pandemic sample (2018–2020), participants implemented the false interview task. (b) In the earlier post-pandemic sample (2020–2022), participants executed both the false interview task and the PED task. (c) In the later post-pandemic sample (2022–2023), participants completed the PED task and then filled in the questionnaires.

## Results

### Comparing data pre- and post-COVID-19 outbreak

The analyses below were conducted on data from participants’ performance on the false interview task sampled during the pre-pandemic and the earlier post-pandemic stages (2018–2020 and 2020–2022, respectively). These analyses included only comparisons of the false interview task, since this was the only task shared by the two samples.

#### Distance preference (Fig. 3A)

**Figure 3:**
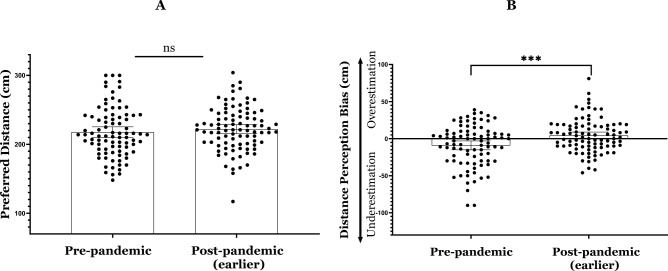
Mean differences and 95% confidence intervals of data collected in the pre-pandemic phase and the earlier post-pandemic phase (two years before and after the outbreak) for: (**A**) Distance preference. (**B**) Distance perception.; * =*p* < 0.05, ** =*p* < 0.01, ** * =*p* < 0.001.

To prospectively examine changes in distance preference following COVID-19, we conducted an independent t-test, with *year* (pre-pandemic and the earlier post-pandemic) as an independent variable and *preferred distance* as a dependent variable. We hypothesized that year would exhibit a main effect, such that preferred distance would be at the earlier post-pandemic. The difference preference model did not reach significance *t*_(176)_ = − 0.874, *Cohen’s d* = − 0.131, *p* = 0.192. Specifically, differences in participants’ preference for distance after the COVID-19 outbreak (*M* = 222.04, *SD* = 31.93) did not differ from their distance preference before the outbreak (*M* = 217.58, *SD* = 36.06). Further, note that in all the analyses described below, BF01 was calculated for all non-significant findings, based on previous literature that used Bayesian factor in post-hoc analysis for null hypothesis significance testing (NHST)^48^. A Bayesian analysis yielded BF_01_ = 2.698, indicating that the null model is 2.698 times more likely than the main-effect model. Furthermore, note that for all analyses we calculated achieved power using post-hoc analysis in G*Power software (version 3.1.9.7)^49,50^. Results indicated an achieved power of 0.49 (*t*_(174)_ = 0.872).

#### Distance perception (Fig. 3B)

To prospectively examine changes in distance perception following COVID-19, we conducted an analysis similar to the one described above, with distance perception bias as the dependent variable. We had no hypothesis concerning the main effect of year or for the interaction; therefore these parts were exploratory. The distance perception model was significant *t*_(176)_ = − 3.446, *Cohen’s d* = 0.517, *p* = 0.001. Specifically, participants' interpersonal distance estimates in the earlier post-pandemic phase (*M* = 4.10, *SD* = 22.71) were greater than their pre-pandemic distance perceptions (*M* = − 9.12, *SD* = 28.10).

### Changes in distance preference and perception: examining the influence of time since the COVID-19 outbreak and R value

The following analyses were conducted on the PED task results for participants sampled during the earlier post-pandemic phase (2020–2022). In these analyses, we examined changes in distance preference and perception as a function of *time elapsed since COVID-19 outbreak* and *R Value*. For purposes of replication, in the later post-pandemic sample we conducted these analyses on the number of days since the outbreak. Note that the 2023 analyses did not include the R value measure because the Israel Ministry of Health had stopped collecting these data.

#### Time since outbreak

##### Distance preference (Fig. 4A)

**Figure 4:**
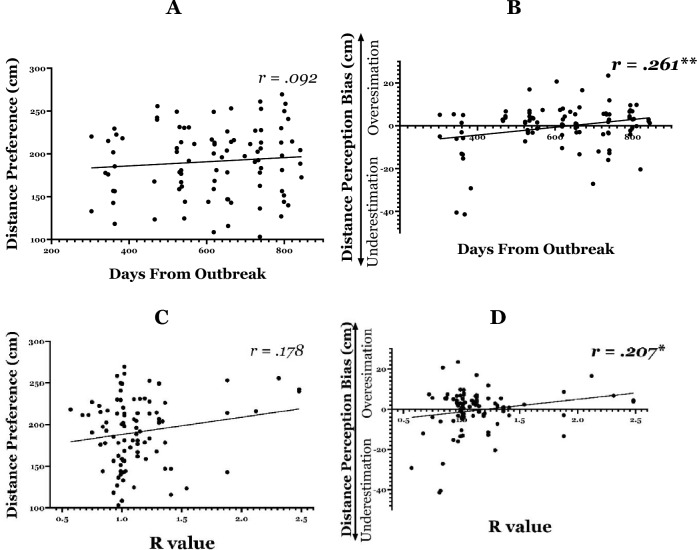
Change in interpersonal distance as a function of objective risk: (**A**) Correlation between distance preference and number of days elapsed since the outbreak of the pandemic in Israel. The results show that preferred distance did not significantly change as the COVID-19 crisis evolved. (**B**) Correlation between distance perception bias and number of days elapsed since the outbreak of the pandemic in Israel. The positive correlation indicates that as the COVID-19 crisis evolved, individuals perceived strangers to be farther away than they actually were. (**C**) Correlation between distance preference and R value. The results suggest that people’s preferred distance did not significantly change as the infection rate increased. (**D**) Correlation between distance perception bias and R value. The positive correlation indicates that as the infection rate rose, strangers were perceived as farther away than they actually were.

A Pearson correlation was calculated with *preferred distance* in the PED task as the dependent variable and number of *days since the outbreak* as independent variable. We hypothesized that objective risk would be positively correlated with distance preference, such that as the COVID-19 crisis evolved participants would show a preference for maintaining a greater distance from strangers. No significant correlation was found between the number of days since the outbreak and the distance preference (*r* = *0.0*92, *R*^2^ = 0.008*, p* = *0.7*78), indicating that the time elapsed since the start of the outbreak had no effect on the distance participants preferred to maintain from strangers (Fig. 4A). A Bayesian analysis yielded BF_01_ = 3.266, indicating that the null model is 3.266 times more likely than the main-effect model. Furthermore, post-hoc power analysis for Bivariate normal model indicated that the achieved power was 0.94 (*Critical r* = 0.08).

##### Distance perception (Fig. 4B)

The distance perception analysis was similar to the analysis described above. Because this is the first study to examine distance perception in COVID-19, the analysis was exploratory. A significant positive correlation was found between the number of days since the outbreak and the distance perception bias (*r* = *0.261*, *R*^2^ = 0.068*, p* = *0.001*), indicating that a greater amount of elapsed time since the first COVID-19 outbreak predicted an overestimation bias in assessing interpersonal distance, such that strangers appeared to be farther away than they actually were (Fig. 4B).

#### R value

##### Distance preference (Fig. 4C)

**Figure 5:**
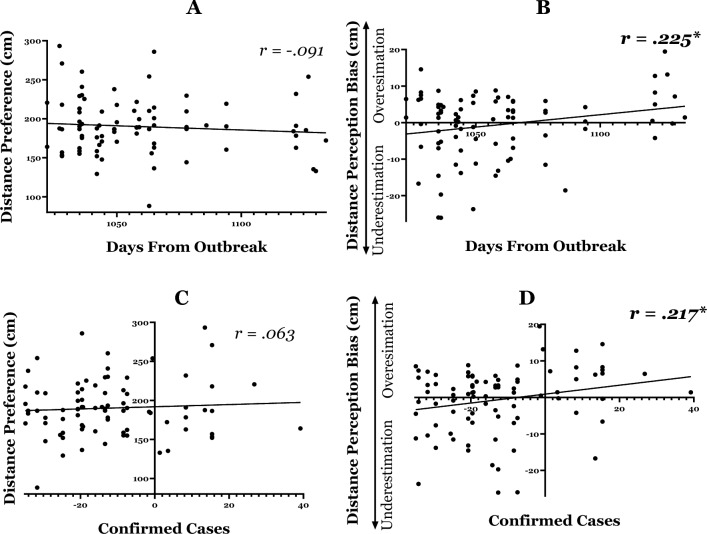
Replication of the findings in the earlier post-pandemic phase for time and risk: (**A**) Correlation between distance preference and number of days elapsed since the outbreak of the pandemic in Israel. The results show that preferred distance did not significantly change as the COVID-19 crisis evolved. (**B**) Correlation between distance perception bias and number of days elapsed since the outbreak of the pandemic in Israel. The positive correlation indicates that as the COVID-19 crisis evolved, individuals perceived strangers to be farther away than they actually were. (**C**) Correlation between distance preference and confirmed cases. The results suggest that people’s preferred distance did not significantly change as the confirmed cases increased. (**D**) Correlation between distance perception bias and confirmed cases. The positive correlation indicates that as the confirmed cases rose, strangers were perceived as farther away than they actually were.

A Pearson correlation was calculated with *preferred distance* in the PED task as the dependent variable and number of *R value* as independent variable. We hypothesized that objective risk would be positively correlated with distance preference, such that as the risk of infection increased participants would show a preference for maintaining a greater distance from strangers. No significant correlation was found between the number of days since the outbreak and distance preference (*r* = *0.1*78, *R*^2^ = 0.032*, p* = *0.0*92), indicating that the R value had no effect on the distance participants preferred to maintain from other people (Fig. 5A). A Bayesian analysis yielded BF_01_ = 1.281, indicating that the null model is 1.281 times more likely than the main-effect model. Furthermore, post-hoc power analysis indicated that the achieved power was 0.64 (*Critical r* = 0.142).

##### Distance perception (Fig. 4D)

A significant positive correlation was found between the number of days since the outbreak and the distance perception bias (*r* = *0.207*, *R*^*2*^ = 0.043*, p* = *0.048*), indicating that a higher R value predicted a bias toward overestimating interpersonal distance, such that strangers appeared to be farther away than they actually were (Fig. 5B).

#### Correlation between time and R value

To examine whether the R value and the number of days since the outbreak share the same variance in COVID-19, we calculated a Pearson correlation between the two factors. We hypothesized that the two measures were not correlated, suggesting that they represent two distinct variances. The results indicate that the R value and the number of days since the outbreak were not correlated (*r* = *0.048*, *R*^*2*^ = 0.002*, p* = *0.650*), such that the peaks in the infection rate occurred at different time points and did not change linearly over time. Post-hoc power analysis indicated that the achieved power was 0.78 (*Critical r* = 0.041).

#### Correlation between the PED task and the false interview task

To examine whether the predictive values of the two measures correlate, we calculated two Person correlations between the preferred distance scores, and between the perception bias scores. The results indicated a correlation between the tasks in the preferred distance scores (r = 0.236, R^2^ = 0.056, *p* = 0.012), while the correlation between the perception scores was not significant (r = 0.049, R^2^ = 0.002, *p* = 0.322).

### Testing replicability in the later post-pandemic phase

The following analyses were conducted on the PED task results of participants in the later post-pandemic phase (2022–2023).

#### Time since outbreak

##### Distance preference (Fig. 5A)

No significant correlation was found between the number of days since the outbreak and distance preference (*r* = − *0.0*91, *R*^2^ = 0.008*, p* = *0.2*05), indicating that the time elapsed since the start of the outbreak had no effect on the distance participants preferred to maintain from strangers. A Bayesian analysis yielded BF_01_ = 3.264, indicating that the null model is 3.264 times more likely than the main-effect model. Furthermore, post-hoc power analysis indicated that the achieved power was 0.49 (*Critical r* = 0.09).

##### Distance perception (Fig. 5B)

A significant positive correlation was found between the number of days since the outbreak and the distance perception bias (*r* = *0.225*, *R*^2^ = 0.051*, p* = *0.020*), indicating that more time elapsed since the COVID-19 first outbreak predicted a bias toward overestimating interpersonal distance, such that strangers appeared to be farther away than they actually were.

#### Confirmed cases

##### Distance preference (Fig. 5C)

No significant correlation was found between coronavirus confirmed cases and distance preference (*r* = − *0.0*63, *R*^2^ = 0.004*, p* = *0.2*83), indicating that the time elapsed since the start of the outbreak had no effect on the distance participants preferred to maintain from strangers. A Bayesian analysis yielded BF_01_ = 6.243, indicating that the null model is 6.243 times more likely than the main-effect model. Furthermore, post-hoc power analysis indicated that the achieved power was 0.50 (*Critical r* = 0.06).

##### Distance perception (Fig. 5D)

A significant positive correlation was found between coronavirus confirmed cases and the distance perception bias (*r* = *0.217*, *R*^2^ = 0.047*, p* = *0.024*), indicating that increase in confirmed cases predicted a bias toward overestimating interpersonal distance, such that strangers appeared to be farther away than they actually were.

#### Correlation between time and confirmed cases

The results indicate that confirmed cases and the number of days since the outbreak were not correlated (*r* = *0.062*, *R*^2^ = 0.004*, p* = *0.572*), such that the increase in the confirmed cases occurred at different time points and did not change linearly over time. A Bayesian analysis yielded BF_01_ = 6.272.

### COVID-related anxiety and its relationship with distance preference and perception

The following analyses were conducted on the PED task results of participants in the later post-pandemic phase (2022–2023).

#### Distance preference (Fig. 6A)

**Figure 6:**
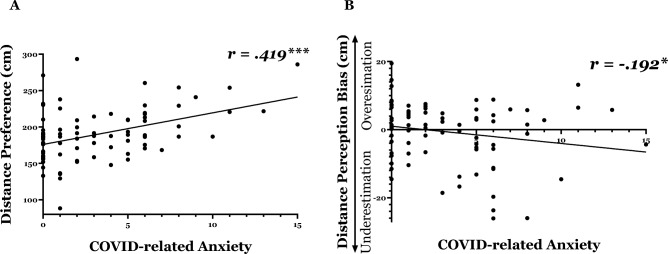
Change in interpersonal distance as a function of COVID-related anxiety: (**A**) Distance preference: As anxiety levels increased, participants displayed a preference for maintaining a greater interpersonal distance. (**B**) Distance perception: As anxiety levels increased, participants tended to underestimate interpersonal distance, perceiving others as closer than they actually were.

We conducted a Pearson correlation, with the *COVID-related anxiety score* as an independent variable and the *preferred distance* in the computerized measure as the dependent variable. Based on the literature, we hypothesized that the *COVID-related anxiety* score would be positively correlated with distance preference, such that participants would prefer to maintain a greater distance from strangers as their COVID-related anxiety level increased. A significant positive correlation was found between CAS score and distance preference (*r* = *0.419*, *R*^2^ = 0.175*, p* < *0.001*), indicating that higher COVID-related anxiety predicts a preference for maintaining a greater interpersonal distance.

#### Distance perception (Fig. 6B)

The analysis was similar to the analysis described above, with distance perception bias as a dependent variable. Based on our previous studies on anxiety and interpersonal distance perception (i.e., Givon-Benjio et al., 2020; Givon-Benjio & Okon-Singer, 2020) and on the literature, we hypothesized that the CAS score would be negatively correlated with distance perception bias, such that highly anxious participants would underestimate the distance. A significant negative correlation was found between CAS score and the distance perception bias (*r* = − *0.192*, *R*^2^ = 0.037*, p* = *0.040*), indicating that higher COVID-related anxiety predicts a bias toward underestimating interpersonal distance, such that strangers appeared to be closer than they actually were.

#### Generalized anxiety and its relationship with distance preference and perception

We conducted the analyses described above with the GAD score as the independent variable. No correlation was found between the GAD-7 score and distance preference (*r* = *0.218*, *R*^2^ = 0.047*, p* = *0.063; Power* = 0.67), indicating that generalized anxiety did not predict distance preference. Furthermore, no correlation was found between the GAD-7 score and distance perception bias (*r* = − *0.116*, *R*^2^ = 0.013*, p* = *0.326; Power* = 0.72), indicating that generalized anxiety did not predict distance perception.

#### Correlation between COVID-related anxiety and GAD

We tested the correlation between COVID-related anxiety and GAD to examine whether they share the same variance. A significant correlation was found between COVID-related anxiety and GAD-7 scores (*r* = *0.337*, *R*^2^ = 0.114*, p* = *0.003*), indicating that individuals with high COVID-related anxiety also experienced high levels of generalized anxiety.

## Discussion

### Results summery

The results of the current study demonstrate that objective risk of COVID-19 infection is associated with a change in the way people perceive interpersonal distance, but not with a change in the distance individuals actually maintain from one another. Specifically, participants demonstrated a bias toward overestimating interpersonal distance, such that other people were perceived as farther away than they actually were. Nevertheless, and contrary to what we expected and to the official policy and distance restrictions, the physical interpersonal distance people maintained during the pandemic was the same as their interpersonal distance before the pandemic, such that they did not change their distance preference in the face of objective risk (i.e., infection rate and confirmed cases). Furthermore, the distance preference did not change as a function of time, indicating that there were no slower changes in the distance people preferred to maintain as time progressed. These results were obtained and replicated in three different samples and in two tasks, highlighting the reliability of the results. Yet COVID-related anxiety was associated with contradictory results. Specifically, COVID-related anxiety was associated with perceiving other people as closer than they actually are and also predicted a preference for maintaining greater distance from others. Despite strong correlations with generalized anxiety, only COVID-related anxiety predicted the bias in perception, suggesting that this cognitive bias is specifically associated with the pandemic. Taken together, the combined results suggest that COVID-19 has generated a long-term change in the way we visually perceive interpersonal distance and may shed light on the processes underlying public compliance to the restrictions.

### COVID-19 changed the way distance is perceived, not the distance actually maintained

We propose two mechanisms, influenced by subjective and objective risk, to account for these findings. To begin with, concerning objective health-related risk (infection rate), we found that individuals did not significantly alter the physical distance they maintained from others. However, their perception of this distance shifted after the onset of the pandemic, making them perceive others as being farther away. This shift can be understood as an overestimation bias acting as a compensatory mechanism: Given the challenges people face when trying to change their behavior^51^, the impact of COVID-19 on their cognitive processing created an illusion of maintaining a greater interpersonal distance. This interpretation aligns with existing research emphasizing the difficulty of behavior change, even when health-related factors are at stake^52^. Additional support for this idea comes from longitudinal studies showing the stability of behaviors from childhood to adulthood^53^, as well as the limited effectiveness of behavioral interventions^54^. On the other hand, some studies suggest that cognitive processing can be more readily modified. For example, short-term cognitive training can lead to changes in targeted cognitive functions^55^, although it rarely transferred to actual behavior^56^. Therefore, we speculate that adjusting perceived distances obviates the need for behavioral changes, thereby creating dissonance in which people believe they are adhering to the restrictions without actual compliance.

A similar effect of perception on behavior can be observed outside the context of COVID-19. Consider, for instance, the ‘size-speed illusion’^57,58^, in which larger objects, like trains and buses, appear to be moving slower than smaller objects, such as cars, even when traveling at the same speed. Although the public is required to wait for a signal before crossing, the decision to cross a railroad early is influenced by the apparent speed of the train, leading individuals to falsely estimate the time required to clear the crossing. This bias is considered one of the main causes of car-train collisions^57,58^. Therefore, the lack of adherence may be rooted not in a lack of willingness or risk-taking behavior but in distorted visual perception.

### Fear-driven distance perception and preference: the role of COVID-19 anxiety

The second mechanism suggested in this paper is associated with subjective risk. Specifically, COVID-related anxiety predicted people’s actual approach-avoidance behavior, such that highly fearful individuals preferred to maintain greater distance from other people. This finding is in line with previous literature suggesting that distance preference during the COVID-19 pandemic reflected people’s interpretation of the situation as risky, rather than an objective risk^24,59^. In addition, COVID-related anxiety (but not general anxiety) was associated with underestimation of distance, such that highly fearful individuals perceived other people as closer in physical proximity. This finding is in line with previous literature suggesting that personal fear may alter perception of a threatening stimulus by intensifying its threatening elements^60^. For example, individuals with spider phobia tend to overestimate the size of spiders^61–63^, and fear of heights is associated with overestimation of heights^64,65^. In the context of interpersonal distance, previous studies found that social anxiety—which is characterized by a fear of close physical proximity, especially with strangers^66^—was associated with perceiving strangers (but not friends or neutral stimuli) as being closer than they actually are^36,37^. Such perception biases likely play a role in protecting individuals from potential psychological/physical harm by enhancing the apparent danger, therefore promoting avoidance. In the context of the current findings, for individuals with high COVID-related anxiety, physical proximity is threatening (due to fear of infection). Hence, the perception bias may act as a *self-preservation mechanism* by reducing the apparent distance, thus motivating the individual to prefer to maintain a greater distance. Support for this notion also lies in the fact that the distance perception bias was only found to be correlated with COVID-related anxiety levels and not with generalized anxiety (GAD), despite the strong correlation between the two types of anxiety. This finding underscores that this bias occurs only when interpersonal distance poses a threat. This interpretation is also supported by the research of Kühne and Jeglinski-Mende^44^, who found that at close proximity (i.e., 50 and 90 cm) people underestimated interpersonal distance in a third-party perspective. These researchers also interpreted this underestimation as a mechanism that is triggered by fear of infection and that promotes avoidance.

An alternative interpretation of the findings is that following lockdowns, especially during the later phases of the pandemic after vaccines were introduced, people were lonely and might have experienced a desire for greater proximity to others. This increased desire for closeness could have motivated them to perceive others as being closer. This interpretation aligns with previous studies that have demonstrated how desirable objects are perceived as being closer than less desirable ones^33^. In the context of our study, the underestimation of distance could be viewed as a reflection of the motivation to be physically closer to other people. This alternative perspective is also supported by a recent study indicating that COVID-related loneliness predicts a preference for maintaining closer proximity to other people^22^. It is worth noting that interpersonal distance perception has not been explored in the context of loneliness, whether COVID-related or not. Therefore, for a comprehensive understanding of the motivations behind this bias, future studies should investigate the role of loneliness in shaping interpersonal distance perception.

### Limitations

The current study has several limitations. To begin with, the finding of an unchanged distance preference due to COVID-19 was underpowered, with an average statistical power of 67 (ranging from 49 to 94). The literature recommends a power level above 80 (or 90 in some cases) to avoid Type II errors. The Bayesian factor provided moderate support for the null hypothesis (H0), with an average BF0 of 3.350 (ranging from 1.281 to 6.243). These combined analyses suggest that with a larger sample, a significant result could have emerged, indicating that people do indeed maintain a greater distance due to COVID-19. Therefore, the conclusion that COVID-19 did not change the preferred distance should be approached with caution. It is worth noting that despite the limited statistical power, our results have been replicated in different samples and measurements, implying that although we cannot rule out changes in distance preference on a larger sample, such effects are weaker than the impact COVID-19 had on distance perception. However, it is essential to recognize that null results in our study may be influenced by various other factors beyond sample size, such as cultural, psychological, and situational variables^67^.

In relation to the previous limitation, there are notable inconsistencies with previous studies. To begin with, prior research demonstrated a preference for greater distance during COVID-19^23–27^. However, due to limited statistical power, it is possible that a similar effect would have been observed in the current study with a larger sample. Nonetheless, it’s important to note that our findings align with Iachini and colleagues^24^, who suggested that people maintain a greater interpersonal distance only in the face of subjective risk, not actual risk. Within this framework, the inconsistency observed may stem from a fear of being infected with COVID-19, especially in the early stages of the pandemic. Future studies should further investigate the role of subjective versus objective risk to clarify this point. Another inconsistency arises with Kühne and Jeglinski-Mende^44^, who demonstrated an underestimation of short distances and an overestimation of long distances. In the current study, however, the perception bias was calculated as an average of randomly chosen distances. Additionally, Kühne and Jeglinski-Mende^44^ focused on perception biases in a third-person perspective (e.g., observing two other people from the side), whereas the current study examined the perceptual distance from the self. Therefore, this discrepancy could be attributed to differences in task design.

Additionally, data were collected at three distinct time points (pre-pandemic, earlier post-pandemic, and later post-pandemic), with different sets of participants at each time point. As such, the study does not follow a longitudinal design and, therefore, does not provide information on the individual changes in distance preference and perception as the pandemic unfolded. Moreover, when testing the correlation between the computerized measure (PED task) and the ecological measure (the false interview task), only the preference scores were correlated, but not the perception scores. Interpretation of this finding could be that for the perception bias, the two tasks do not tackle the same construct and, therefore, should not be considered as measures of the same phenomenon. Other explanations for this difference include task differences; for example, the false interview task may elicit more fear due to its realistic nature, making socially anxious individuals more sensitive to it. Additionally, the false interview task is based on a single trial, whereas the PED task includes multiple trials, suggesting that the result of the PED task may be more stable in the perception measure. Nevertheless, it is important to approach the interpretation of replicated results with a degree of caution.

In addition, in the pre-pandemic sample, participants already preferred a distance exceeding 2 m. This suggests that the non-significant difference in distance preference following the outbreak could stem from a pre-existing inclination aligning with pandemic restrictions. However, it's important to note that the infection rate in Israel did not align with the expected lower rates under this assumption. Additionally, considering the confined space of the interview room in the false interview task and the early realization that 2 m might not suffice in close spaces, participants were likely to maintain a greater distance than 2 m to avoid infection. It is more likely that the preference for a distance greater than 2 m in the pre-pandemic sample was influenced by elements in the task design, such as the anxiety associated with the expectancy of being interviewed.

Furthermore, the current design does not rule out alternative cognitive mechanisms. One possibility is that biased estimation results from a distortion in perceptual processes, as suggested by previous studies that demonstrated alterations in visual perception due to motivational states^35,68^ and emotional states^69^. However, in the current study, the stimulus was not present at the time of judgment, making it challenging to exclude other potential mechanisms. For instance, the current findings might be attributed to a memory bias, with participants remembering others as being farther away, possibly because they expected this to be the case. It is worth noting that participants made their judgments only a few milliseconds after viewing the video clip (PED task) and 1–2 min after chair placement (false interview task). This makes it less likely that a memory bias is the cause. Nevertheless, there is also evidence suggesting that cognitive or high-level factors cannot directly affect perception^70^. Therefore, future studies should aim to examine the specific mechanism underlying this anomaly while carefully controlling for alternative explanations.

### Conclusions

The current study examined changes in interpersonal distance preference and perception following the COVID-19 outbreak. Since the data were collected during the critical period of the COVID-19 pandemic, it offered a unique opportunity to explore interpersonal distance preference and perception under major restrictions on social distance. Our findings illuminate how perception adapts to public restrictions, offering valuable insights for policy development and refinement.
